# Regional Mortality Disparities in Germany: Long-Term Dynamics and Possible Determinants

**DOI:** 10.1007/s11577-015-0329-2

**Published:** 2015-09-21

**Authors:** Eva U. B. Kibele, Sebastian Klüsener, Rembrandt D. Scholz

**Affiliations:** 1 Population Research Centre, Faculty of Spatial Sciences; Healthy Ageing, Population and Society (HAPS), University of Groningen, Groningen, The Netherlands; 2 Max Planck Institute for Demographic Research, 18057 Rostock, Germany

**Keywords:** Regional mortality, Mortality trends, Socioeconomic conditions, (Eastern and western) Germany, Regionale Sterblichkeit, Sterblichkeitstrends, Sozioökonomische Bedingungen, (Ost- und West-)Deutschland

## Abstract

While regional mortality inequalities in Germany tend to be relatively stable in the short run, over the course of the past century marked changes have occurred in the country’s regional mortality patterns. These changes include not only the re-emergence of stark differences between eastern and western Germany after 1970, which have almost disappeared again in the decades after the reunification of Germany in 1990; but also substantial changes in the disparities between northern and southern Germany. At the beginning of the twentieth century, the northern regions in Germany had the highest life expectancy levels, while the southern regions had the lowest. Today, this mortality pattern is reversed. In this paper, we study these long-term trends in spatial mortality disparities in Germany since 1910, and link them with theoretical considerations and existing research on the possible determinants of these patterns. Our findings support the view that the factors which contributed to shape spatial mortality variation have changed substantially over time, and suggest that the link between regional socioeconomic conditions and recorded mortality levels strengthened over the last 100 years.

## Abstract (de)

Regionale Unterschiede in der Sterblichkeit in Deutschland erweisen sich kurzfristig betrachtet als relativ stabil. Im Laufe des letzten Jahrhunderts wurden allerdings deutliche Veränderungen in den regionalen Sterblichkeitsmustern verzeichnet. Hierzu zählt nicht nur das Wiederauftreten ausgeprägter Ost-West-Unterschiede ab 1970, die in den Jahrzehnten nach der deutschen Wiedervereinigung 1990 fast wieder verschwunden sind. Vielmehr haben sich auch substanzielle Verschiebungen zwischen Nord- und Süddeutschland ergeben. Zu Beginn des 20. Jahrhunderts registrierten die norddeutschen Regionen die höchsten Lebenserwartungsniveaus, während die süddeutschen Landesteile die niedrigsten verzeichneten. Über die letzten 100 Jahre hat sich dieses räumliche Muster weitgehend umgekehrt. In diesem Beitrag analysieren wir die langfristigen Entwicklungen der räumlichen Mortalitätsunterschiede in Deutschland seit 1910. Die beobachteten Veränderungen setzen wir in Bezug zu theoretischen Überlegungen und existierenden Forschungsergebnissen zu möglichen Ursachen für regionale Mortalitätstrends. Unsere Ergebnisse zeigen auf, dass sich die Zusammensetzung der maßgeblich zur Entwicklung der regionalen Sterblichkeitsmuster beitragenden Faktoren über die Zeit erheblich verändert hat. Außerdem scheint sich der Zusammenhang zwischen regionalen sozioökonomischen Bedingungen und den registrierten Sterblichkeitsniveaus in den letzten 100 Jahren verstärkt zu haben.

## Background

Since 1910, German society has undergone a number of fundamental developmental processes which have led to substantial improvements in the survival chances of its individual members. This rapid rise in life expectancy has been achieved by many societies across the globe, and is sometimes referred to as the longevity revolution. At the beginning of the twentieth century, large parts of German society could still be characterised as agrarian. However, rapid industrialisation in the second half of the nineteenth century had already spurred urban growth. Particularly in big cities, governments struggled to alleviate the poor hygienic conditions which contributed to an urban penalty in survival chances (Woods 2003). Infectious diseases were the primary causes of death, and infant mortality was high. Today, on the other hand, the death of an infant has become a rare event, and cardiovascular diseases and cancer have emerged as the leading causes of death.

These shifts have also contributed to a growing gap in the survival chances of people based on their social status (Bengtsson and Van Poppel 2011). In spatiotemporal contexts with unfavourable hygienic conditions and a high prevalence of infectious diseases, individuals of higher socioeconomic status face difficulties in translating their better access to resources into improvements in survival chances. But socioeconomic status may be more relevant in contexts in which deaths are mainly caused by non-communicable diseases, as the occurrence of these diseases can be influenced by health-related behaviours, and mortality risks can be reduced through resource-intensive medical treatments. Thus, we might expect the survival chances of individuals in highly developed societies to be much less affected by uncertainties and randomness, and to be much more dependent on their access to assets (e.g., human and financial capital). As a result, regional variation in socio-economic development might today be more strongly linked to regional variation in mortality disparities than in earlier periods.

Germany is well suited for an investigation of regional mortality differences, as it appears to have been the European country with the highest degree of sub-national mortality variation in the early years of the twentieth century (Klüsener et al. 2014). Germany was long divided into dozens of independent states which differed in terms of their economic structures and their prevailing religious and social norms. Even today, Germany has a highly federalised structure, which is apparent in the organisation of the health care and educational systems. The country’s location in central Europe, at the crossroads of Western and Eastern Europe, makes Germany a particularly interesting case to study. Europe has long been characterised by substantial east-west differences in mortality conditions (Vallin and Meslé 2001), which are also reflected to some extent in Germany’s regional mortality patterns.

Many studies on variation in longevity have focused on the national level, e.g., in the form of cross-country comparisons (Meslé and Vallin 2002; Nolte et al. 2000a; Omran 1998; Vallin and Meslé 2001). However, national averages might hide substantial variation across sub-national regions of a country (Bonneux et al. 2010; Shaw et al. 2000). In some cases, these national means might even paint a misleading picture. For example, if a country exhibits high levels of infant mortality in the north and low levels in the south, the mean national levels will not be representative of either of the two parts of the country. Studies with sub-national data allow us to look into such regional discrepancies. In addition, investigating how regional variation patterns have changed over time is likely to improve our understanding of the potential determinants which facilitated or limited the longevity revolution at different periods of time. These factors might include spatial variation in the compositional characteristics of populations or of contextual circumstances, such as socioeconomic and ecological conditions.

In recent decades, a large number of studies have examined regional mortality differences in Germany. However, most of these studies have focused on disparities between East and West Germany after the division of the country in 1945, or on regional mortality differences at some point in time after 1990 (Cromm and Scholz 2002; Kibele 2012; Luy 2004; Vogt 2013). Our study broadens this focus not only by taking a longer-term perspective over a whole century, but also by providing a higher degree of spatial detail. The use of this approach allows us to explore variation within eastern and western Germany. To our knowledge, this is the first study which has taken such a long-term view on regional mortality disparities in Germany. Our paper has two aims. The first aim is to provide an overview of the theoretical considerations regarding the factors which have contributed to spatial variation in survival conditions at different stages of the longevity revolution. The second aim is to compare these theoretical models with descriptive evidence on the development of regional mortality differences within Germany.

## Theoretical considerations

Theoretical and conceptual models which seek to explain regional mortality differences are scarce in the demographic literature, and there are even fewer theoretical models which attempt to explain changes in regional mortality differences over time. Most of the scholars who have engaged in the discourse on explanatory models are in the fields of health sciences (Kawachi and Berkman 2003; Lalonde 1974), geography (Gatrell 2002), or both (Shaw et al. 2002). Our theoretical considerations benefit considerably from Lalonde’s (1974) health field concept, which focuses on mortality disparities; and from Omran’s (1998) epidemiologic transition theory, which seeks to explain mortality decline over time.

### Explaining regional mortality variation by population composition and regional context

In the 1970s, the Canadian Department of Health and Welfare developed the health field concept, which acknowledged the important role that non-medical factors play in health, and provided a classification of these factors (Lalonde 1974). The concept was originally developed to depict and explain health disparities, and to support policy interventions (cf. Kickbusch 2003). It envisaged that the health field can be broken down into four elements: human biology, the physical and social environment, individual lifestyle, and the health care system (Lalonde 1974). In line with Lalonde, we also assume that regional mortality disparities originate from both macro-level conditions and regional variation in individual-level characteristics and behaviour. Individual-level determinants of mortality can influence regional mortality variation if the compositional characteristics of populations in terms of individual attributes vary across regions. For example, there may be variation in the share of university graduates or in the share of smokers. Regional differences might be reinforced through social interactions in which spatial distance can moderate the frequency and intensity of interactions between individuals; e.g., a higher prevalence of smokers in an area might increase the likelihood that other people who live in the area will start to consume tobacco products. Other macro-level influences, which are also referred to as contextual effects, include spatial variation in socioeconomic, political, and ecological conditions (Diez Roux 2002; Shaw et al. 2002; Valkonen 2001).

Over the past two decades, one of the key issues raised in the scientific debate on spatial variation in health and survival outcomes has been how best to disentangle compositional and contextual effects. Scholars who have addressed this question have often used multilevel models (Diez Roux 2002; Kawachi and Berkman 2003; Riva et al. 2007; Tunstall et al. 2004). These models attempt to distinguish influences at different levels of aggregation (e.g., individuals nested in regions and/or countries). Empirical findings based on multilevel models suggest that while individual-level characteristics determine to a large extent the lifespans of individuals living in modern societies, there are also independent contextual effects on mortality. Mortality is usually higher among individuals who live in areas with less favourable socioeconomic conditions (Meijer et al. 2012). This seems to be particularly the case among people of lower socioeconomic status (Kibele 2014; Riva et al. 2007; Tunstall et al. 2004).

#### Population composition: individual-level mortality determinants

At the individual level, age and gender are strong mortality predictors. Therefore, mortality measures usually standardise for differences in age structure, while the calculations are done separately for men and women. We also follow this strategy in our study. Age is an important factor, not only because of the biological accumulation of health risks and diseases over an individual’s life course, but also because social factors, such as social inclusion and access to assets, can vary systematically across the lifespan. Likewise, gender differences in mortality risks are related to both biological and social factors (Luy 2004). Genetics play a major role in determining individual’s mortality risks (Christensen and Vaupel 1996). Nevertheless, genetic shifts appear to have contributed very little to the massive improvements in survival chances among European societies during the longevity revolution of the last two centuries (Burger et al. 2012). Thus, while studies have found that similarities in genetic characteristics are decreasing by spatial distance across Europe (Lao et al. 2008), the role of genetics in determining regional variation in the longevity revolution is probably small.

The elements of an individual’s lifestyle which are generally referred to as health behaviours—such as nutrition, physical activity, and smoking and drinking habits—are strongly and directly related to mortality risks. These health behaviours differ regionally. Variation was observed during the time when Germany was divided, as East Germans had more limited access to healthy foods (Diehl 2008; Nolte et al. 2000b). Lifestyle and health behaviours are strongly correlated with socioeconomic status, which is another important survival determinant at the individual level. The socioeconomic position of an individual within a society not only influences the person’s lifestyle, but also has an independent effect on his or her mortality. Thus, a person’s socioeconomic position is usually strongly linked to his or her access to financial assets, such as income and savings; human capital, including education and health status; and infrastructure capital, such as good housing (Shaw et al. 2002). Spatial variation in occupational characteristics can also contribute to disparities in mortality outcomes. For example, mining regions have a high share of miners, who have elevated mortality risks (Tunstall et al. 2004). Access to social capital (Bourdieu and Wacquant 1992), which may, for example, affect access to informal care and social inclusion at older ages, can also vary across regions.

Selective migration can substantially influence the regional population composition, as regions and cities which offer more job opportunities are particularly attractive in-migration destinations for people both within and outside of the country. Migration is often health-selective: i.e., migrants tend to be healthier than stayers (Boyle 2004), although this effect may be reversed when migrants reach older ages (Kibele and Janssen 2013).

#### Regional context: macro-level mortality determinants

Our considerations on the macro-level mortality determinants in the social and spatial contexts in which individuals are embedded are based on a relational conceptualisation of space (see Cummins et al. 2007 for a detailed discussion). According to this relational view, the spatial location attributes of individuals are not constant over time, but are instead subject to systematic and unsystematic changes at various temporal scales (e.g., daily routines or life course-related migration strategies). The frequency and the quality of an individual’s contacts with other people, and his or her access to assets, societal institutions, or elements of the infrastructure (e.g., the health care system), may be moderated by the absolute geographic distances between the location of the individual and the location of other people and objects. The relevance of absolute geographical distance does, however, vary across space and individuals, depending on the availability and accessibility of transport and communication infrastructures. This also implies that the importance of absolute geographic distances has been substantially reduced over our study period as a result of innovations in transport and communication technologies, and the development of related infrastructures. Some macro-level determinants may be related to discrete spatial areas (e.g., specific regional policies), while others may vary in a fluid manner across space, and thus have no discrete boundaries. Some factors can fluctuate substantially across small areas (e.g., neighbourhoods), while others might exhibit more variation at lower levels of spatial detail (e.g., bigger regions within a country). There may also be associations between individual attributes and contextual conditions; e.g., the health effects of living in a deprived area might be moderated by socioeconomic status.

The degree to which the administrative territorial divisions we refer to in studying variation in life expectancies are relevant for understanding the macro-level determinants also depends on the social relevance of these divisions. While Germany was divided, the border between eastern and western Germany was of considerable relevance, as it separated the country into two states with little social interaction and with very different political and economic systems. But also the administrative division of the country in 1910 was mainly based on previously long-standing borders between independent German states. The social, economic, and political characteristics of these states varied substantially. Even today, Germany has a decentralised political structure in which the federal states *(Bundesländer)* play a role in organising the health care infrastructure and in formulating regional education and economic development policies. There is also substantial variation in socioeconomic conditions across the German federal states.

Contextual macro-level factors include variation in socioeconomic and ecological conditions and (regional) policies, and spatial variation in access to infrastructure such as housing and health and emergency care (see also Diez Roux 2002). Particularly for historical patterns, variation in religious norms and in the extent to which mothers breastfeed their infants also affect mortality outcomes (Van Poppel 1992). In regions with unfavourable social and economic conditions, a culture of anomie might emerge (Shaw et al. 2002). This culture may foster the spread of unhealthy lifestyles. Access to health infrastructure might also exhibit a centre-periphery gradient, as relative to residents of peripheral areas, people living in big cities may have better access to specialised hospitals and shorter rescue times in case of health emergencies.

### Explaining mortality decline and regional differences in mortality decline

Given the large number of determinants of regional mortality and the complexity of these influences, it is not surprising that explaining *changes* in regional mortality differences is difficult (Valkonen 2001). Changes in regional mortality variation could be attributable to regional trends in risk factors at both the individual and the contextual levels. The importance of various mortality determinants may fluctuate over time, and changes in risk factors are translated into disease patterns, often with a temporal delay (Kuh et al. 2004; Valkonen 2001). We use Omran’s epidemiologic transition theory as a guideline for explaining changes in (regional) mortality patterns over time (Omran 1998). Over our study period of 1910 until today, Germany transitioned from being at the end of the second stage of *receding pandemics* (characterised by a decrease in the frequency of epidemics, limited access to health care and sanitation, and improvements in housing conditions); to the third stage of *degenerative, stress, and man-made diseases* (characterised by the greater prevalence of cardiovascular and other degenerative diseases and broad access to preventive and curative health care); and finally to the fourth stage of *declining cardiovascular mortality, ageing, lifestyle modification, and emerging and resurgent diseases*. The fourth stage started with the cardiovascular revolution in the 1970s (Vallin and Meslé 2001). It is characterised by sustained chronicity and ageing, deliberate modifications in health-related lifestyles, and health care interventions designed to address the risk factors of chronic diseases (Omran 1998).

At the beginning of the twentieth century, infectious diseases were the most important cause of mortality. But as the prevalence of these diseases sharply declined, there were significant reductions in infant, child, and maternal mortality rates (Omran 1998). Public health measures related to hygiene and nutrition resulted in increased living standards. In particular, the installation of water mains and sewage disposal systems led to a decline in mortality (Omran 1998; Vallin and Meslé 2001). In Germany, sewage and drinking water purification systems were first introduced in cities at the start of the industrial revolution, from 1850 to 1880. There is, however, little empirical evidence of a direct link between the reduction in infant mortality and these public health measures (Van Poppel and van der Heijden 1997; Wolleswinkel-van den Bosch 1998). The regions that industrialised early usually did not provide healthier living environments. Instead, the working conditions at the onset of industrialisation led to increases in mortality due to hard physical labour, exposure to toxic substances, and accidents (Wolleswinkel-van den Bosch 1998).

Most medical innovations—such as antibiotics, surgical innovations, and perinatal care—became effective after 1930, when infectious disease mortality had already declined significantly due to social advances and hygiene (Omran 1998; Wolleswinkel-van den Bosch 1998). However, there seems to be a stronger link between medical advancements and mortality improvements during the cardiovascular revolution that started in the 1970s (Vallin and Meslé 2001). Innovations in medical care contributed substantially to improvements in survival chances at older ages. The longevity revolution has also been fostered by a range of socioeconomic, political, and cultural factors—including lifestyle choices related to health, nutrition, and living standards—as well as by medical and public health advances. The effects of each of these factors on mortality have also changed over time (Omran 1998; Vallin and Meslé 2001).

## Analytical strategy and data

### Analytical strategy

In the empirical part of this paper, Germany’s regional mortality patterns and trends are described and discussed in relation to our theoretical considerations and the potential determining factors discussed in the literature. We first present trends in life expectancy at birth for Germany between 1910–1911 and 2009–2011 (Statistisches Bundesamt 2012a, 2012b). Changes in life expectancy over time are decomposed into age group-specific contributions in order to show which age groups in particular benefited from improvements at different time periods (Preston et al. 2001). Between 1945 and 1990, eastern and western Germany are distinguished.

We use cartographic analyses for the investigation of variation in regional life expectancy. The regional period life tables were calculated separately for men and women to derive life expectancies at birth (Preston et al. 2001). Life expectancy at birth expresses the number of years a newborn can expect to live under the survival conditions around the time of birth; i.e., if the mortality rates at different ages did not change over time. Regional life expectancies at birth for men and women are displayed in maps. For each map the derived regional life expectancy levels are classified into five colour categories according to a standard deviation classification centred on the mean. Category boundaries are set at 0.5 and 1.5 standard deviations above and below the mean.

Among the challenges we faced in conducting our analysis of long-term regional mortality in Germany is the fact that both the external and the internal administrative boundaries of the country changed several times over the past 100 years. Particularly drastic were the changes in eastern Germany, as the regional divisions which were established in the GDR era differed substantially from those which existed prior to 1945. The administrative divisions implemented in eastern Germany after 1990, however, are again much closer to those in place before 1945. In western Germany, by contrast, there has been a relatively high degree of temporal continuity in administrative boundaries, at least at the level of the federal states. As our paper is a descriptive account of how mortality differences developed over time, these changes in administrative divisions are not of immediate concern for our analysis. Nevertheless, the colour categorisation schemes in the maps can be affected by the changing number of regions in Germany over time. For example, the eastern regions of the German Empire generally lagged behind the western regions in the longevity revolution. The loss of these regions in 1945 most likely reduced the overall variance in regional life expectancy. Consequently, the life expectancy differences in the remaining parts of Germany would have appeared more pronounced in the maps even if the regional life expectancies in these parts had not changed.

### Data

We present data on regional mortality variation for the following periods: 1910, 1970, and 1995–2011. These time periods were selected based on theoretical and data availability considerations. The year 1910 marks the end of the rather peaceful period following the unification of the German Empire in 1871. During these decades, the country underwent rapid economic development, and a national welfare system emerged. For the 1910 observation period we obtained age-group specific mortality rates for the states and territories of the German Empire covering the years 1908–1913. These data also allowed us to subdivide the biggest German state, Prussia, into its provinces; and the second-biggest state, Bavaria, into parts east and west of the Rhine (Statistisches Reichsamt 1918; *N* = 40). Based on these data, we calculated regional life expectancies at birth.

We decided not to collect data for the rather turbulent time period between the beginning of World War I in 1914 and the erection of the Berlin Wall in 1961, during which Germany experienced two wars and substantial migration streams from east to west. Instead, the time period around 1970 was chosen as the second observation period. This time period marks the beginning of the emergence of distinct differences in mortality between East and West Germany (Luy 2004). It was also approximately the time when the southern part of West Germany became the most economically advanced area in West Germany. The data for the West German regions (NUTS 1; *Bundesländer; N* = 11) for the period 1970–1972 were obtained from regional life tables calculated by the Federal Statistical Office (Statistisches Bundesamt 2012a). The life expectancy data for the East German regions (*Bezirke; N* = 15) in 1968–1971 were derived from regional life tables calculated by the Statistical Office of the GDR (Ministerrat der DDR—Staatliche Zentralverwaltung für Statistik 1973).

To account for the effects of the reunification of Germany in 1990 and the large mortality changes in eastern Germany which followed, we provide more detailed mortality data at the district level (NUTS 3; *Kreise*) for the period 1995–2011. As the colour categorisation schemes in the maps can be affected by changes in the number of districts, we created a dataset of regional units with time-constant boundaries for this period by combining all of the districts which exchanged territories as a result of reforms in district-level borders (*N* = 396). Life expectancy was calculated from the age-specific population and death counts (using data provided by the statistical offices of the federal states; Statistische Ämter des Bundes und der Länder 2014). Unfortunately, due to a number of far-reaching reforms in the administrative boundaries of the eastern German districts in the period 1990–1994, it was not possible to include these years in our district-level analysis of mortality trends.

The GIS shapefiles for the maps were derived from the MPIDR Population History GIS Collection (MPIDR 2014), which offers a series of annual shapefiles with information on the administrative division of Germany over the last 200 years.

## Results

### Trends in life expectancy 1910–2010: an increase of more than 30 years

**Fig. 1:**
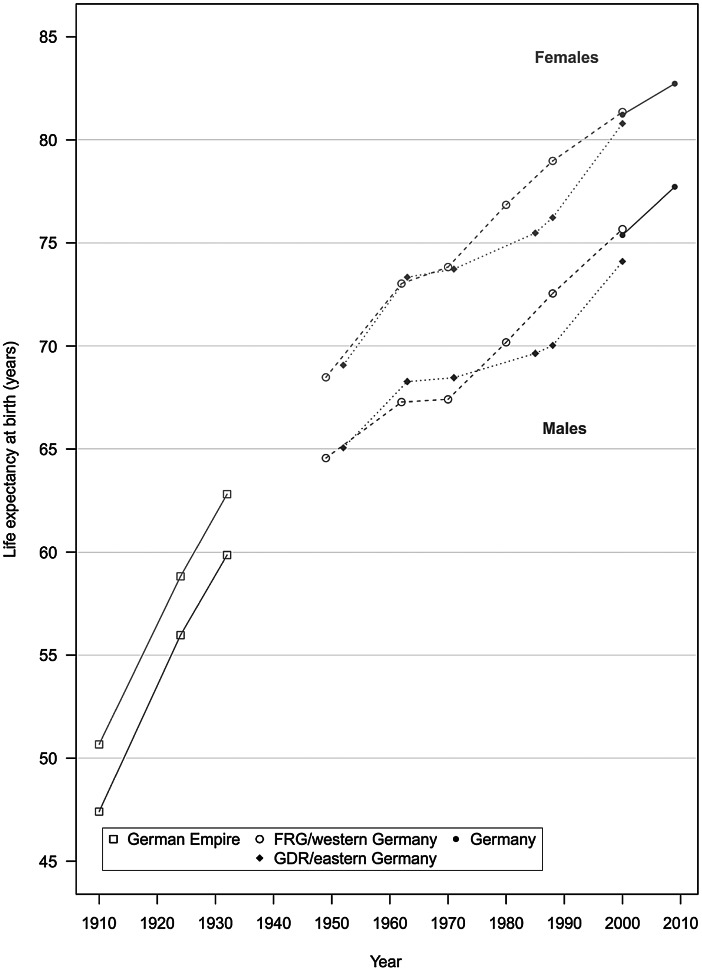
Life expectancy in Germany between 1910–1911 and 2009–2011. (Source: Life table calculations are based on population and death counts obtained from Statistisches Bundesamt 2012a, b). Data from 1910–19 11 to 1932–1934 refer to the German Empire. Data from the Federal Republic of Germany (FRG) refer to West Germany, including West Berlin, from 1949–19 51 to 1988–1990; and to western Germany, excluding West Berlin, for 2000–2002. Data for the German Democratic Republic (GDR) refer to East Germany, including East Berlin, from 1952–19 53 to 1988–1989; and to eastern Germany, excluding East Berlin, for 2000–2002. Data for Germany for the last period from 2000–20 02 to 2009–2011 refer to Germany as a whole

**Fig. 2:**
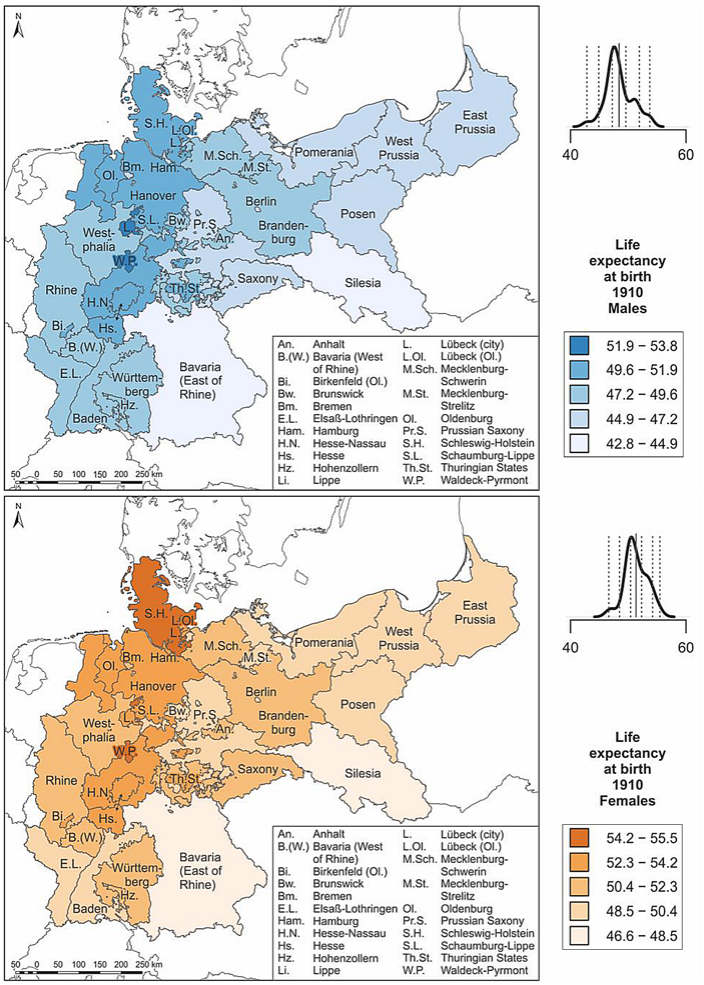
Life expectancy around 1910. (Source: Life table calculations are based on population and death counts obtained from Statistisches Reichsamt 1918. Base map: MPIDR  2014). The rates have been calculated using population data from the census of 1910. At that time, enumeration standards had not yet been completely harmonised throughout the German Empire. Most German states counted the *de facto* (present) population, but Saxony and the Hanseatic cities of Hamburg, Lübeck, and Bremen reported the *de jure* (registered) population (Lee and Schneider 2005). Distortion effects resulting from this deviation are likely to be small, but may exist in both directions. The plot above the legend displays the density curve. In this graph the solid vertical line indicates the mean value, while the dotted vertical lines show the category breaks (based on a standard deviation classification centred on the mean)

**Fig. 3:**
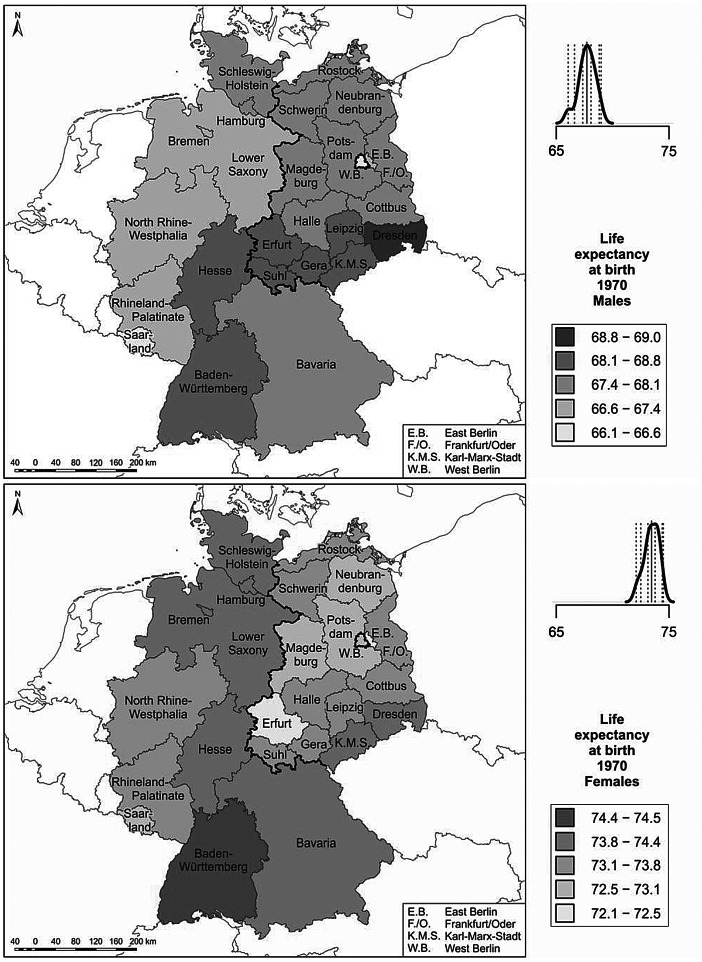
Life expectancy around 1970. (Source: Life tables were calculated by Statistisches Bundesamt 2012a; Ministerrat der DDR—Staatliche Zentralverwaltung für Statistik 1973. Base map: MPIDR 2014). Life expectancy values are shown for the West German *Bundesländer* for the period 1970–1972, and for the East German *Bezirke* for the period 1968–1971. The plot above the legend displays the density curve. In this graph the solid vertical line indicates the mean value, while the dotted vertical lines show the category breaks (based on a standard deviation classification centred on the mean)

**Fig. 4:**
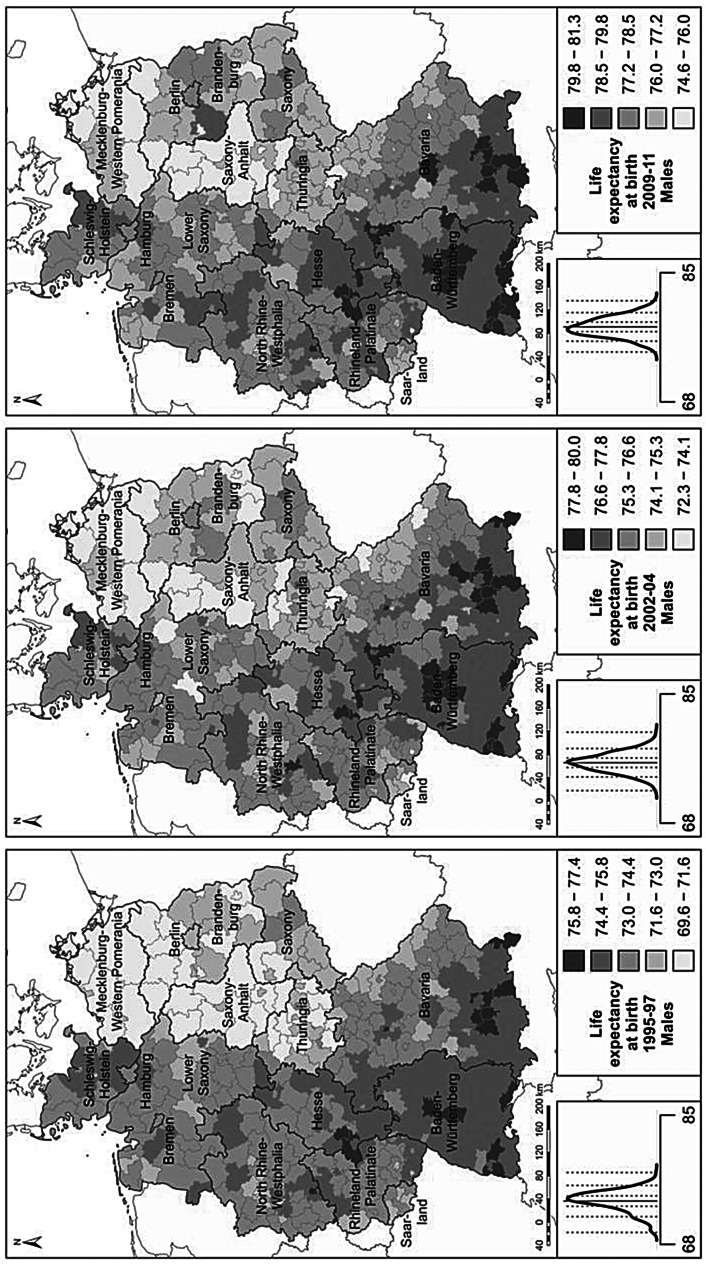

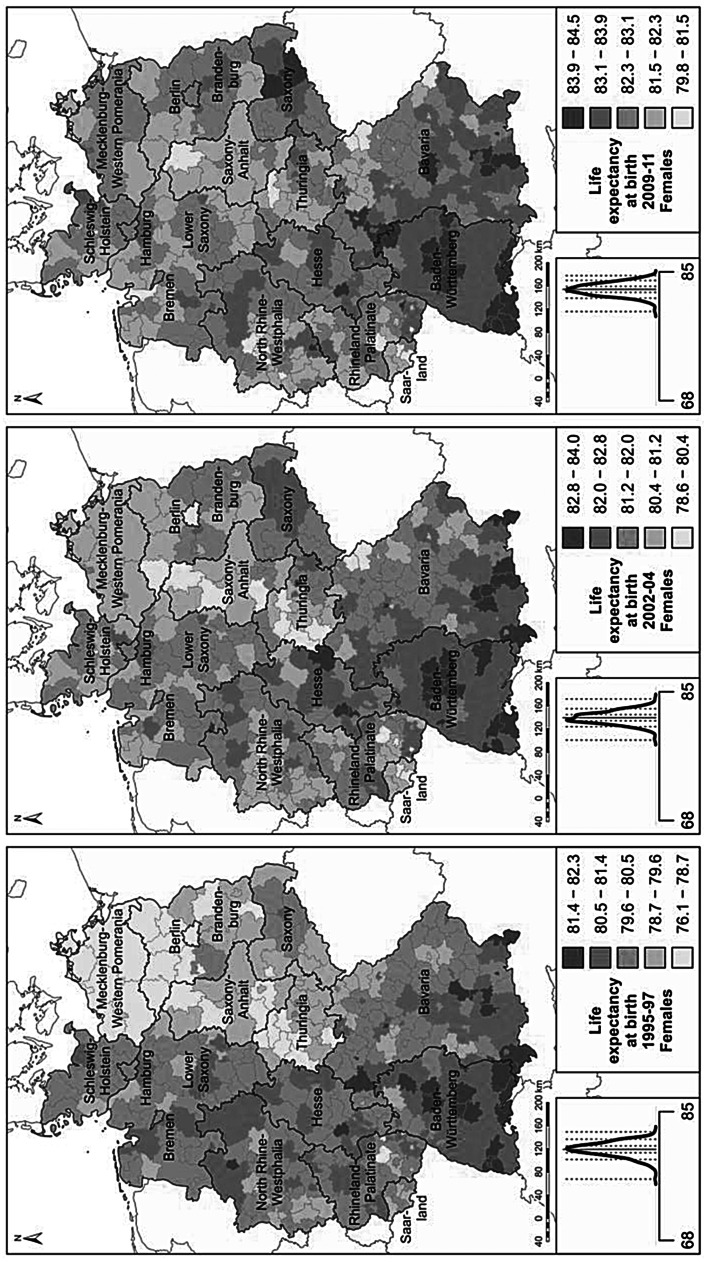
Life expectancy in 1995–1997, 2002–2004, 2009–2011. (Source: Life table calculations are based on population and death counts obtained from statistical offices of the federal states; Statistische Ämter des Bundes und der Länder 2014. Base map: MPIDR 2014). The plot left of the legend displays the density curve. In this graph the solid vertical line indicates the mean value, while the dotted vertical lines show the category breaks (based on a standard deviation classification centred on the mean)

Between 1910 and 2010, life expectancy at birth in Germany increased from 47.4 to 77.7 years among men and from 50.7 to 82.7 years among women (Table 1). Table 1 shows the life expectancy changes for approximately every decade from 1910 until 2011 (depending on data availability). Life expectancy trends between two succeeding time periods are decomposed into age groups, indicating the proportion a specific age group contributed to the life expectancy change. Whereas since the start of the cardiovascular revolution in the 1970s life expectancy improvements mainly occurred among the older age groups, the table shows that in the first half of the century the biggest improvements were predominantly clustered among the younger age groups. At the national level, women had higher life expectancies at birth than men throughout the study period (see Fig. 1). As our subsequent analyses of regional variation will show, regional disparities tended to be smaller among women than among men in all three observation periods. Generally, however, the regional mortality patterns developed in a very similar manner among both men and women (Figs. 2, 3, 4).

**Table 1:** Decomposition of trends in life expectancy at birth (LE) between 1910–1911 and 2009–2011 by age groups; contributions are shown as a percentage of the overall change in life expectancy. (Source: Life table calculations are based on population and death counts obtained from Statistisches Bundesamt 2012a, b)

Time period	German Empire			FRG/western Germany					GDR/eastern Germany							Germany
	1910–1911– 1924–1926 1924–1926– 1932–1934			1949–1951– 1962–1964 1962–1964– 1970–1972 1970–1972– 1980–1982 1980–1982– 1988–1990 1988–1990– 2000–2002					1952–1953– 1963–1964 1963–1964– 1971–1972 1971–1972– 1985–1986 1985–1986– 1988–1989 1988–1989– 2000–2002							2000–2002– 2009–2011
*Men*																
LE at the beginning of the time period	47.4	56.0		64.6	67.3	67.4	70.2	72.5	65.1	68.3	68.5		69.6	70.0		75.4
LE at the end of the time period	56.0	59.9		67.3	67.4	70.2	72.5	75.7	68.3	68.5	69.6		70.0	74.1		77.7
Average annual change in LE	0.59	0.49		0.21	0.02	0.28	0.30	0.26	0.29	0.02	0.08		0.13	0.33		0.26
*Age group*	Decomposition: contribution of age groups to changes in life expectancy (in %)															
0	48.6	50.2	79.9		230.0	33.1	14.4	8.7	61.1	454.5	50.4		35.7		8.7	2.2
1–14	21.6	14.7	17.5		45.3	8.6	5.5	3.5	12.4	84.2	13.4		18.2		3.9	1.8
15–29	3.0	16.3	8.8		− 78.9	10.0	11.2	5.0	3.0	45.1	11.4		23.5		3.3	8.5
30–44	8.1	5.2	10.4		− 46.0	7.2	8.5	8.2	6.3	− 46.4	3.4		− 19.4		7.5	10.7
45–59	10.9	5.6	3.8		26.4	7.5	19.1	17.5	3.9	53.7	− 13.3		− 18.8		20.8	15.9
60–74	6.4	6.7	− 19.9		− 54.0	26.9	27.8	34.0	− 0.8	− 223.1	31.7		25.3		35.8	36.8
75 +	1.5	1.3	− 0.5		− 22.9	6.6	13.4	23.2	14.2	− 268.0	3.1		35.4		20.0	24.1
*Women*																
LE at the beginning of the time period	50.7	58.8	68.5		73.0	73.8	76.9	79.0	69.1	73.3	73.7		75.5	76.2		81.2
LE at the end of the time period	58.8	62.8	73.0		73.8	76.9	79.0	81.4	73.3	73.7	75.5		76.2	80.8		82.7
Average annual change in LE	0.56	0.50	0.35		0.10	0.30	0.27	0.20	0.39	0.05	0.13		0.25	0.36		0.17
*Age group*	Decomposition: contribution of age groups to changes in life expectancy (in %)															
0	46.9	42.8	41.3		30.9	26.0	15.2	8.1	38.2	194.0		25.3	15.2	5.9		4.1
1–14	25.4	15.3	10.4		5.4	6.2	4.8	3.2	9.4	37.1		7.8	1.8	2.7		2.2
15–29	5.6	15.2	9.2		− 1.7	4.0	5.3	2.9	7.7	18.6		5.4	4.7	2.0		4.3
30–44	7.3	10.9	7.9		8.7	5.8	5.1	6.7	7.6	37.4		7.6	3.0	5.1		8.0
45–59	5.8	6.7	8.8		3.3	12.9	13.6	8.0	5.4	30.5		13.6	8.8	14.5		11.7
60–74	6.9	7.3	15.1		39.6	22.2	25.3	32.0	13.1	− 43.6		23.0	27.2	36.4		29.1
75 +	2.1	1.8	7.3		13.8	22.9	30.6	39.0	18.5	− 173.9		17.2	39.4	33.5		40.6

Data from 1910–19 11 to 1932–1934 refer to the German Empire. Data from the Federal Republic of Germany (FRG) refer to West Germany, including West Berlin, from 1949–1951 to 1988–1990; and to western Germany, excluding West Berlin, for 2000–2002. Data for the German Democratic Republic (GDR) refer to East Germany, including East Berlin, from 1952–1953 to 1988–1989; and to eastern Germany, excluding East Berlin, for 2000–2002. Data for Germany for the last period from 2000–20 02 to 2009–2011 refer to Germany as a whole

Percentage values above 100 in the decomposition outcomes are particularly likely to occur if strong improvements in mortality in an age group are happening in periods with small changes in overall life expectancy. For example, life expectancy at birth for women in the GDR increased by just 0.39 years between 1963–1964 and 1971–1972. Strong declines in infant mortality contributed to an increase in life expectancy at birth of 0.76 years; this increase was, however, counteracted by mortality increases at higher age groups, as indicated by the negative values

### Life expectancy around 1910: high life expectancy in the north

In presenting our results related to regional mortality variation in Germany at different points in time, we first describe for each period the variation pattern displayed in the map. This is followed by a discussion of potential determinants of the regional pattern, which we derive from our theoretical considerations and the existing literature. Figure 2 shows regional variation in life expectancy at birth for men and women at our earliest observation period of around 1910. The regions with comparatively high life expectancies were at that time concentrated in the northern and central parts of present-day Germany. These regions include Schleswig-Holstein, Mecklenburg, parts of present-day Lower Saxony and Hesse, as well as some territories in Thuringia. Regions with very low life expectancies include Bavaria in the south-eastern part of present-day western Germany and Silesia in the south-eastern part of the German Empire.

Most of the existing literature which has examined the factors that may have contributed to the regional variation in life expectancy at the beginning of the twentieth century has focused on infant mortality and its determinants. This is not surprising given that life expectancy at birth is highly sensitive to deaths at very young ages, which were still frequent at that time. Thus, the spatial variation in life expectancy at birth was to a large extent determined by the variation in infant mortality. As a consequence, the regional infant mortality patterns and the regional life expectancy patterns were broadly similar (for maps of infant mortality, see Klüsener et al. 2014). In 1910 in the German Empire, there was a considerable degree of regional variation in infant mortality levels, with an average of 181 deaths below age one per 1000 live births among males and of 152 deaths per 1000 live births among females. Many areas in north-western and central Germany had fewer than 100 infant deaths per 1000 live births. These numbers were close to those of the adjacent Scandinavian countries, which in this period were the European vanguard countries in the longevity revolution. By contrast, in several parts of Bavaria more than 300 infants per 1000 live births did not survive their first year of life. These levels were among the highest recorded in Europe at that time, and were similar to the levels found in eastern and south-eastern European countries such as Russia and Hungary (Klüsener et al. 2014).

Research by Kintner (1987) has suggested that spatial variation in the prevalence of breastfeeding is relevant for understanding the spatial patterns in infant mortality. In north-western Germany almost all children were breastfed. In Bavaria, by contrast, relatively few infants were breastfed. Instead, there was a long-standing practice in Bavaria of feeding newborns with meal pap and other artificial foods. This resulted in a high prevalence of water-borne diseases such as diarrhoea, which can cause infant death (Kintner 1987). As breastfeeding also supports the immune systems of infants, the mortality risks of adults who were not breastfed as children might have also been affected (see Klüsener and Scholz 2013). In the two decades following World War I, a spatial convergence in breastfeeding patterns in Germany took place. In the 1930s, for example, almost all of the children in Bavaria were breastfed, a change which may be attributable to information campaigns on the benefits of breastfeeding (Kintner 1988). Parallel to these developments, spatial variation in infant mortality levels almost disappeared. A direct causal link between the two trends could not, however, be established (Kintner 1988). In addition, improvements in access to purified water might have contributed to a decrease in the negative effects of feeding infants with artificial foods.

Variation in economic development levels may have also affected the life expectancy patterns. At the beginning of the twentieth century, the landlocked regions of southern Germany were lagging behind other German regions in terms of economic development. Compared to the southern regions, the northern and central regions of Germany had better access to harbours, and thus benefited earlier from rapidly developing global markets (see also Klüsener and Zagheni 2014). In additional analyses presented in Appendix 1, we investigated the potential link between regional variation in gross domestic product (GDP) per capita and regional disparities in life expectancy at birth for the period around 1910. Across all regions for which GDP data were available, a weak positive relationship with life expectancy was found. However, the two city regions of Berlin and Hamburg constitute outliers, as they recorded by far the highest GDP per capita levels, but only average life expectancy levels. This finding might be interpreted as an indication that the urban mortality penalty discussed above was still relevant at that time; i.e., that the effects of the higher levels of economic development in these big cities, which might be expected to lead to higher average life expectancy levels, were offset by poor hygienic conditions and pollution in big cities. After we excluded these two city regions from the analysis, the positive relationship between economic development and life expectancy at birth was much stronger (see Appendix 1).

In addition to breastfeeding practices and economic conditions, climatic factors might have played a role in the regional mortality patterns, as seasonal and annual variation in temperature and precipitation increased from the maritime north-western parts to the more continental southern and south-eastern parts of Germany. Thus, the southern regions were more susceptible than the northern regions to extreme weather conditions, such as dry periods and cold and heat waves, which may in turn also have affected harvest variability and temporal variation in local access to food.

### Life expectancy between 1910 and 1970: from strong increases to stagnation

As is the case in other European countries, more than half of the increase in life expectancy of more than 30 years between 1910–1911 and 2009–2011 in Germany was achieved in the first 50 years of the study period (Fig. 1; Vallin and Meslé 2001). The life expectancy gains in the first half of the twentieth century were attributable to very large declines in infant and child mortality, and to a much lesser extent to improvements in mortality at middle and older ages (Table 1). Advances in the treatment of infectious diseases and hygiene, including the use of antibiotics starting in the 1940s, were the main drivers of mortality declines in this period (Omran 1998; Vallin and Meslé 2001).

We were able to investigate trends in regional infant mortality rates between 1910 and 1940 (own analyses based on data from the Statistical Yearbooks of the Germany Empire; analyses not shown). This investigation demonstrates that around 1910, (north-)western German regions such as Hanover and Hesse-Nassau still had much lower infant mortality rates than Bavaria in southern Germany and eastern German regions such as Mecklenburg-Schwerin and the Prussian province of Saxony (the territories of which are today mainly in Saxony-Anhalt). However, the infant mortality rates of these regions rapidly converged in the 1920s and 1930s. Remarkably, the biggest improvements appear to have occurred in 1918–1930, a post-war period which was characterised by very unfavourable economic conditions, including hyperinflation in 1923 and the onset of a world economic crisis.

After World War II and the subsequent division of Germany into the capitalist West Germany (Federal Republic of Germany, FRG) and the communist East Germany (German Democratic Republic, GDR), both states initially reported very similar mortality improvements (Table 1). Among other causes, infectious diseases still played a prominent role in overall mortality in the late 1940s, but the effects of these diseases were greatly reduced in both parts of Germany after 1950 (Vallin and Meslé 2001). During the 1960s, however, both states experienced a period of stagnation in which hardly any life expectancy gains were achieved (Fig. 1). Decreases in infant and child mortality were partly offset by mortality increases at older ages due to cardiovascular diseases (Table 1; Nolte et al. 2000a).

The national averages for West Germany are, however, misleading, as the trends varied substantially at the subnational level. According to the Federal Statistical Office, between 1960–1962 and 1970–1972 the registered increase in life expectancy in the southern states of Baden-Württemberg and Bavaria and in the centrally located state of North Rhine-Westphalia was about 1 year among males and between 1.5 and 2 years among females. The situation was very different in the three northern states of Hamburg, Bremen, and Lower Saxony (no data are provided for the northern German state of Schleswig-Holstein in 1960–1962). Male life expectancy at birth was decreasing in these three states, and the declines were particularly large in Bremen and Lower Saxony. While women in the north of West Germany also experienced a rise in life expectancy of around 1 year, this increase was far lower than the increase recorded in southern Germany (Statistisches Bundesamt 2006). As economic conditions were good in all of the West German regions at that time, these conditions cannot explain the diverging life expectancy trends. It is also unlikely that these developments were strongly linked to selective migration from northern to southern Germany, as these trends can be observed not only for life expectancy at birth, but also for life expectancy at age 60. As migration mostly occurs at younger ages, life expectancy trends at age 60 and above generally are not affected by selective migration. However, research outcomes on spatial variation in mortality patterns at a higher level of spatial detail provide evidence that economic conditions played a role in shaping mortality patterns at that time. Kuhn et al. (2006) have argued that the current regional mortality patterns in Bavaria evolved in the 1960s, mainly along regional economic development axes.

### Life expectancy around 1970: on the verge of the cardiovascular revolution

Figure 3 displays male and female life expectancy levels around 1970 for the federal states *(Bundesländer)* of West Germany (the FRG) and at the level of the regions *(Bezirke)* of East Germany (the GDR). This period approximately marks the beginning of the onset of the cardiovascular revolution in western Europe (Meslé and Vallin 2002). Among men, the highest life expectancy levels were registered in the southern part of the GDR (southern Saxony and Thuringia), while the lowest levels were recorded in West Berlin and the two heavily industrialised areas of North Rhine-Westphalia and Saarland in West Germany. Among women, almost all of the areas with rather low life expectancy levels were in East Germany. West Berlin, which belonged to the FRG, also had low life expectancy. Another mortality hot spot in West Germany was Saarland in the southwest. The highest female life expectancy levels throughout the two German states were recorded in the south of the FRG, particularly in Baden-Württemberg; and in the far north. Thus, clear-cut life expectancy disparities between East and West Germany were not discernible in the early 1970s, at least among men.

In the literature, the regional mortality differences in the period around 1970 were mainly attributed to spatial variation in medical care and environmental pollution (Gatzweiler and Stiens 1982; Vogt 2013). At that time, infant mortality—which is often thought to reflect the quality of medical care—still played a role in regional variation in life expectancy (Table 1). The FRG appears to have had higher infant mortality levels than the GDR around 1970. However, this discrepancy may be related to differences in reporting practices (Luy 2004). A study initiated by the GDR government which looked at variation in infant mortality at the district level *(Kreise)* in the GDR in 1966 concluded that the differences in infant mortality rates were related to the quality of the paediatric care (Institut für Planung und Organisation des Gesundheitsschutzes 1968).

Elevated pollution levels in highly industrialised regions may have affected the short- and long-term health of all of the inhabitants of these regions. In addition, these regions tended to have higher shares of workers in hazardous occupations, which might have contributed to the elevated regional mortality levels. In the GDR, a higher burden of emissions was associated with higher mortality, and particularly with cardiovascular mortality (Vogt 2013). In the FRG, excess mortality was found in the high-density coal and steel industry regions of the Ruhr (the central part of North Rhine-Westphalia) and Saarland. Excess mortality in the industrialised regions stemmed from cancer of the respiratory organs, diseases of the digestive system, bronchitis, and liver cirrhosis. This provides some evidence of a relationship between environmental pollution and elevated mortality risks (Gatzweiler and Stiens 1982).

The elevated levels of mortality found in less industrialised areas in the 1970s—such as in several rural and remote districts in the FRG, and in the northern GDR *Bezirke* which today make up Mecklenburg-Western Pomerania—might be related to social and economic problems in these regions (Gatzweiler and Stiens 1982). In 1970 there was a positive link between the economic development of a region and its pollution levels, which in turn affected the general relationship between regional economic development and life expectancy levels. For example, North Rhine-Westphalia was a leader in terms of GDP per capita, but lagged behind other regions in terms of life expectancy (own analysis based on data from the Federal Statistical Office).

### Life expectancy 1970–1990: emergence of the east-west life expectancy gap

In the early 1970s life expectancy levels in western and eastern European countries started to diverge, and a similar east-west gap began to appear in Germany (Meslé and Vallin 2002; Vallin and Meslé 2001; Fig. 1). The emergence of an east-west life expectancy gap, and its subsequent reduction since the 1990s, are issues that have been frequently studied and discussed (Carlson and Hoffmann 2011; Diehl 2008; Luy 2004; Nolte et al. 2000a). In West Germany there was a strong rise in life expectancy between 1970 and 1990 due to falling mortality in almost all age groups and causes of death. This trend was in large part attributable to substantial reductions in infant mortality and in mortality related to cardiovascular diseases among older people and fatal injuries among young adults (Table 1; Nolte et al. 2000a). A look at the shares specific age groups contributed to life expectancy changes in this period shows that women in East and West Germany had very similar patterns (Table 1). There were, however, large disparities between the two states in terms of the overall improvements achieved. This was also the case among men. Among East German men aged 30–59, mortality improved very little (cf. Carlson and Hoffmann 2011).

Most of the existing studies which attempted to explain the emergence of the east-west life expectancy gap attributed it primarily to regional differences in the development of health care and economic conditions, as well as to lifestyle and environmental conditions (Diehl 2008; Luy 2004; Myrskylä and Scholz 2013). Meslé and Vallin (2002, pp. 182–183) noted that the communist regimes relied heavily on “the centralised administration of modern health care, whereas the struggle against cardiovascular diseases, unlike that against infectious diseases, requires important changes in individual behaviour and the active participation of citizens in the management of their own health care.” In addition, reducing mortality from cardiovascular diseases requires substantial investments in the health care system and medical technologies, for which the GDR government lacked the necessary resources. As a result, the GDR health care system—like the systems in the other state socialist countries—was quite efficient in tackling infectious diseases and reducing the mortality of infants and children in the early years of the GDR, but it could not compete with the West German system in reducing levels of cardiovascular mortality (Carlson and Hoffmann 2011; Nolte et al. 2000a). Pollution levels in West Germany first started to decline in the 1960s, partly as a result of the introduction of higher environmental standards, and partly because heavy industries declined in importance. East Germany, on the other hand, did not register substantial reductions in pollution levels until the late 1980s.

It is also worth noting that unlike in the 1960s, the increases in life expectancy in West Germany in the 1970s and 1980s were roughly the same in almost all of the federal states, independent of their level of economic development. Life expectancy increases were around 4–5 years among both men and women (Statistisches Bundesamt 2006). This suggests that national-level processes played a role in these trends. In East Germany, the *Bezirk* Dresden in Saxony continued to have the lowest mortality levels in the 1970s and 1980s. Within East Germany, the regions which had higher mortality initially experienced the largest mortality declines over time (Casper and Herrmann 1990). This was also the case in the West German federal states (Statistisches Bundesamt 2006).

### Life expectancy after 1990: east-west mortality convergence and its impact on regional life expectancy patterns

There were many changes in mortality in Germany following reunification in 1990. We will first describe the mortality trends for eastern and western Germany. This is followed by a discussion of the regional mortality patterns and developments over time, and possible explanations for detected trends. After reunification in 1990, there was a short-term rise in male mortality in eastern Germany. According to Nolte et al. (2000b), this was largely attributable to traffic-related mortality among young men. However, starting in the early 1990s eastern Germans began to catch up to their western German counterparts, with mortality in the eastern German states declining among all age groups and for all major causes of deaths. This trend led to a convergence of the life expectancy levels of eastern and western Germany, which continues up to today (Table 1; Fig. 1). In the mid-1990s the remaining east-west life expectancy differences were mainly attributable to excess cardiovascular mortality among people aged 60 + . Among men, there was also considerable excess mortality at younger ages from external causes of death and diseases of the digestive system. These sources of excess mortality were greatly reduced, particularly during the 1990s (Table 1; Luy 2004; Nolte et al. 2000b).

For the period since 1995, regional life expectancy patterns can be studied at the fine-gridded district level (Fig. 4). In the mid-1990s, east-west differences were still dominant, but as eastern Germany started to catch up, a more nuanced pattern emerged. In western Germany, regions in the southern German states of Baden-Württemberg and Bavaria maintained their position as German vanguard regions throughout the period. Several high-mortality hotspots can also be found in western Germany. These are mostly concentrated in economically disadvantaged area, including Saarland and adjacent areas of Rhineland-Palatinate; parts of the Ruhr area; north-eastern Bavaria; and a few scattered districts in northern Germany, including all of the North Sea harbour towns. Within eastern Germany, Saxony continued to be the region with the highest life expectancy levels, as it was prior to 1990. Among women in Saxony, mortality levels have declined to the levels reported in low-mortality western German regions (Fig. 4). Some of the other areas of eastern Germany with above-average gains were Thuringia and the Brandenburg districts surrounding Berlin, which received substantial in-migration from Berlin due to suburbanisation processes. Less favourable were the developments in Mecklenburg-Western Pomerania and Saxony-Anhalt, where male life expectancy in particular continued to lag behind that of other regions.

Spatial variation in the survival improvements across the eastern German districts was attributable in large part to sharp declines in deaths from heart disease and traffic-related accidents. There were also substantial reductions in cancer mortality among men and in alcohol-related mortality among women (Kibele 2012). In the entire country, regional excess mortality, and gender differences in regional life expectancy patterns, were partly attributable to behaviour-related causes of death, such as lung cancer, traffic accidents, and alcohol consumption. Rural areas had excess mortality at young adult ages due to traffic-related mortality (Kibele 2012).

For the most recent periods, the literature has largely attributed existing regional mortality patterns to regional differences in socioeconomic conditions. From the 1970s to today, virtually all of the aggregate-level studies on regional mortality in western Germany and in post-reunification Germany have found an association between high mortality and several regional conditions, especially socioeconomic deprivation (Brzoska and Razum 2008; Latzitis et al. 2011). The strong role of socioeconomic mortality determinants has also been confirmed by several studies which looked at variation across districts or municipalities within a specific federal state (e.g., Kuhn et al. 2006; Maier et al. 2012). East-west differences in district-level life expectancy could be traced back almost entirely to differences in socioeconomic conditions (Kibele 2012). As differential regional population composition usually explains a share of the variation in regional morbidity, several more recent studies have applied innovative multilevel techniques to disentangle the effects of population composition and context. These studies revealed that an independent effect of regional conditions (mainly of a socioeconomic nature) on health outcomes remains, even after controlling for a large array of individual-level characteristics. This has been shown for self-rated health (Diehl and Schneider 2011), subjective physical health (Voigtländer et al. 2010), subjective overall health (Eibich and Ziebarth 2014), and long-standing illness among resettlers *(Aussiedler)* (Kreft and Doblhammer 2012). For old-age mortality, such regional effects have been identified for western but not for eastern Germany (Kibele 2014).

Studies that have looked at potential contextual factors have found that access to health care institutions might also play a role in regional mortality differences. Although German regulations mandate that all regions have health care of equal quality and accessibility, inequalities in the provision of health care provision have been observed. For ambulatory care, general practitioners (though not medical specialists) are supposed to be distributed equally across the regions (Ozegowski and Sundmacher 2012). In practice, however, the concentration of general practitioners tends to be higher in wealthier areas and cities, even as some regions have severe shortages. Rural areas in eastern Germany are especially likely to have a shortage of physicians (Kopetsch 2010). People who live in cities may also benefit from having access to more specialised care and shorter distances to hospitals. Empirical studies on the (aggregate-level) association between regional mortality and indicators of health care provision have shown that the relationship between regional mortality and the number of physicians or hospital beds per capita is small or even counterintuitive (Kibele 2012; Kuhn et al. 2006; Latzitis et al. 2011). However, when avoidable mortality—i.e., mortality which could have been prevented through timely and efficient health care and health policies—was taken as an indicator of the quality of the health care, a high correlation with life expectancy across the districts was found (Kibele 2012).

Several studies have attempted to explore whether regional variation in environmental pollution is important for understanding regional mortality disparities, but the results have been inconclusive for the most recent decades and at the regional level (Maier et al. 2012; Latzitis et al. 2011). For the eastern German districts, Vogt (2013) found an association between improvements in air pollution after reunification and life expectancy gains, particularly due to declines in cardiovascular and respiratory mortality. Thus, pollution mitigation as a result of deindustrialisation in eastern Germany after 1990 might have helped to close the life expectancy gap between eastern and western Germany. This suggests that, as pollution has declined in both eastern and western Germany, the link between regional socioeconomic conditions and mortality is less distorted by variation in pollution levels.

## Conclusion

In this paper we took a long-term perspective to study regional mortality disparities in Germany, and followed these differences over the course of a full century. Our empirical analysis focused on spatial and temporal variation in life expectancy at birth as an indicator of population health and survival conditions. We were able to identify both continuities and discontinuities in the spatial patterns. A major discontinuity is that northern Germany, and particularly the north-western part of the country, was at the forefront of the German longevity revolution at the beginning of the twentieth century, but had forfeited this position to southern Germany by the end or our study period. Southern Germany, on the other hand, went from being one of the most disadvantaged areas in terms of mortality levels to being the region with the highest life expectancies in Germany. This trend generally appears to correspond with long-term shifts in regional variation in economic conditions. A continuity, at least among women, seems to be that eastern Germany tends to lag behind western Germany; although the southern part of eastern Germany, and particularly Saxony, represents an exception to this general pattern, both in 1970 and in recent years.

The existing research on determinants which might explain regional mortality variation within Germany in different time periods in the twentieth century suggests that over time, there were substantial changes in the factors which contributed to spatial life expectancy disparities. This aspect is in line with the theoretical considerations of Omran (1998). The results of our analysis indicate that a weak positive association between regional variation in economic development and regional mortality disparities already existed at the beginning of the twentieth century. More recent studies have suggested that this relationship substantially strengthened over time. These outcomes are in line with our theoretical considerations, which built upon work by Bengtsson and Van Poppel (2011). Mortality patterns at the beginning of the twentieth century were also influenced by variation in cultural practices. Thus, it appears that cultural differences contributed to a weaker link between economic development and life expectancy levels at that time. In addition, the populations in most of the areas that industrialised early benefited from elevated levels of economic development, but also faced risks related to high levels of pollution and employment in hazardous occupations. It is likely that the substantial reduction in pollution levels and occupational health risks which contributed to elevated mortality risks in highly developed areas in turn led to the strengthening of the association between economic development and life expectancy levels across regions. This shift may have occurred in parallel to a general shift in the influences shaping regional variation away from the dominance of environmental and contextual influences towards the dominance of the health behaviours and social status of individuals (cf. Kuhn et al. 2006). The containment of infectious diseases, which were previously the main causes of deaths, contributed to this general shift, and helped to make survival outcomes socially more selective (Bengtsson and Van Poppel 2011).

Our study has some limitations. First, the descriptive aggregate-level analysis was complicated by changes in the administrative division of Germany over the last 100 years, including the loss of the territories east of the Oder-Neisse line. Thus, for many parts of Germany it was not possible to conduct the analysis based on time-constant areas, which would have allowed us to study regional trend data. In addition, the level of spatial detail at which data could be obtained varied over time, as small-area mortality data were readily available for the most recent decades only. Moreover, as we were working with aggregate-level information, we were not able to look into the role of compositional and contextual effects and the potential influences of selective migration on the observed pattern. Selective migration can reinforce regional mortality patterns by attracting migrants to prosperous regions which already have lower mortality, as migrants are usually healthier (Boyle 2004; Luy and Caselli 2007).

There are a number of potential avenues for future research which would further improve our understanding of long-term regional mortality trends in Germany. As Germany is situated at the centre of Europe, it would be interesting to consider in an extended analysis regional mortality trends in neighbouring countries as well (cf. Shaw et al. 2000). This would enable us to determine whether the German trends in regional mortality variation are part of a larger spatial variation pattern. In addition, such an analysis would allow us to investigate whether mortality improvements in German regions and the adjacent regions in neighbouring countries took place at the same time and at a similar pace, or whether there were substantial differences. As it appears that the regional mortality disparities cannot be fully explained by the compositional characteristics of populations alone, it would also be interesting to perform case studies on regions with distinctive mortality conditions. For example, new insights might be provided by an in-depth study of regions experiencing low mortality despite adverse conditions, or vice versa; or by an exploration of the effect of selective migration through an analysis of individual-level longitudinal data. In addition, researchers should devote more attention to lifestyle factors, which are usually not adequately addressed in the literature on nationwide regional mortality differences due to a lack of data. Moreover, the investigation of various diseases and health outcomes, including wellbeing from a regional perspective, could help us to further improve our understanding of the causal pathways between regional conditions and population health. This kind of analysis could also enable us to better explain why in some cases regional patterns in health outcomes other than mortality (e.g., morbidity) differ from regional mortality patterns (Diehl and Schneider 2011; Eibich and Ziebarth 2014; Kreft 2015). The findings of these studies could be of considerable relevance for the development of public policies related to health and social issues, such as policies dealing with the allocation of future health care investments.

## Figures and Tables

**Fig. 5:**
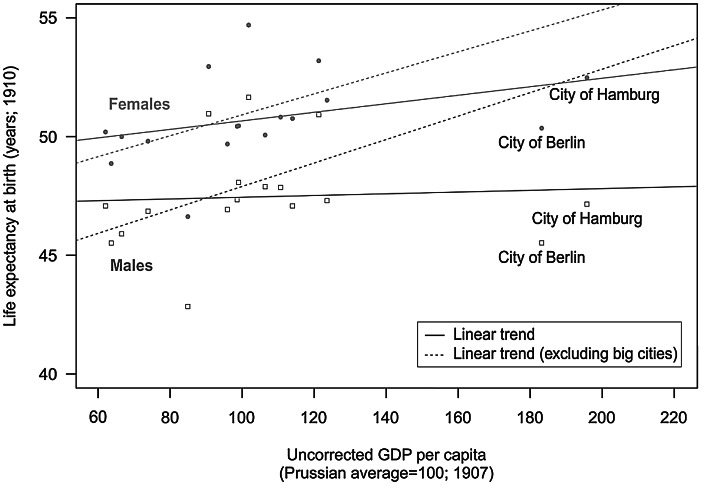
Uncorrected GDP per capita vs. life expectancy at birth around, 1910. (Source: Statistisches Reichsamt 1918; Hoffmann and Müller 1959/2005; own calculations). The German regions covered in this analysis include the Prussian provinces and the German states of Baden, Hamburg, Saxony, and Württemberg (*N* = 18). These territories represented 77 % of the population of the German Empire in 1910. The solid lines show the linear association between GDP and life expectancy for all of the regions; for the dashed line, the two cities of Berlin and Hamburg have been excluded
